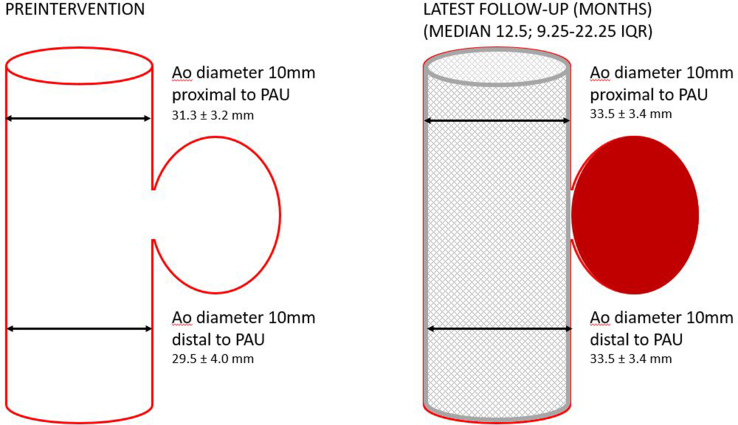# Aortic Remodeling in Patients Undergoing Endovascular Repair for Penetrating Aortic Ulcer With High-Risk Features

**DOI:** 10.1016/j.atssr.2025.07.001

**Published:** 2025-07-29

**Authors:** Pedro J. Furtado Neves, Aline H. Ishida, Emily A. Malgor, Muhammad Aftab, T. Brett Reece, Rafael D. Malgor

**Affiliations:** 1 Division of Vascular Surgery and Endovascular Therapy, University of Colorado, Aurora, Colorado; 2 Division of Cardiothoracic Surgery, University of Colorado, Aurora, Colorado

## Abstract

**Background:**

The natural history of penetrating aortic ulcers (PAUs) with high-risk radiologic features after thoracic endovascular aortic repair (TEVAR) remains incompletely characterized. This study assessed aortic remodeling and midterm outcomes after TEVAR for such lesions.

**Methods:**

We retrospectively reviewed patients undergoing TEVAR for high-risk PAUs between 2016 and 2022. Of 220 TEVAR cases, 12 patients (5.5%) met inclusion criteria with appropriate follow-up. Aortic remodeling was assessed per current societal guidelines.

**Results:**

The cohort included 8 men (66%) with a median age of 73 years and a median follow-up of 31 months (interquartile range, 12.75-59 months). Most PAUs (58%) were located in zone 3; 33% had multiple ulcers, often with >1 high-risk feature. Pre- and post-TEVAR main PAU + intramural hematoma depth averaged 10.1 ± 4.5 mm and 10.1 ± 8.1 mm, respectively. Aortic diameter increased from 41.3 ± 6.0 mm to 45.3 ± 10.4 mm. Complete thrombosis of the main PAU was observed in 92% of patients. Aortic remodeling was positive in 25% and stable in 58% of cases. No 30-day mortality occurred; however, 3 patients (25%) died of nonaortic causes during follow-up. Two reinterventions (17%) were performed for type 2 endoleak and new PAU formation due to cocaine use.

**Conclusions:**

TEVAR for PAUs with high-risk features results in complete thrombosis in most cases. Whereas positive aortic remodeling occurred in a subset, most patients experienced stabilization of aortic dimensions during midterm follow-up.

**In Short**

▪ Thoracic endovascular aortic repair for penetrating aortic ulcers with high-risk features results in favorable aortic remodeling in most cases.
▪ Complete penetrating aortic ulcer thrombosis was observed in 92% of patients, with no 30-day mortality.
▪ Early intervention in selected patients may prevent complications; however, larger prospective studies are needed to define optimal timing and long-term outcomes.

Penetrating aortic ulcers (PAUs), intramural hematoma (IMH), and aortic dissections are different clinical entities within the group of acute aortic syndrome.^1^^,^^2^ As serious and potentially life-threatening conditions, they are often related to advanced atherosclerotic disease. A PAU occurs when the intima and elastic lamina of the aorta are disrupted and the atherosclerotic plaque on the intimal layer of the aorta penetrates into the media.

Reports of PAU natural history in the literature vary, and its management remains controversial.^3^^,^^4^ There have been some data on the natural history of PAU linked to its sequelae.^5^ In addition, data on prognosticators of descending thoracic PAUs and their long-term surveillance with imaging have not been established prospectively, leading to questions about the timing of intervention that has not been determined. Most of the literature pertaining to PAUs located outside of the aorta and those located in the aortic branch vessels relies on large radiology data sets that are queried to determine changes over time or complications related to PAUs.^6^

Recent data have shown that complications are frequent in symptomatic PAUs and IMH, with intervention, recurrent symptoms, radiographic progression, rupture, or death occurring in up to 25% of patients at 30 days and in up to 45% of patients at 1 year.^7^ Currently, management is mainly based on risk factors that have been identified as high-risk features linked to complications demonstrated on imaging studies; these high-risk features suggest that early intervention might be indicated.^4^^,^^8^^,^^9^

One of the areas yet to be explored, however, is how aorta remodeling occurs after thoracic endovascular aortic repair (TEVAR) in patients with high-risk imaging features. The aim of this study, therefore, was to describe aortic remodeling after TEVAR in patients with PAUs and associated high-risk radiologic features.

## Patients and Methods

A retrospective cohort study was performed after patients with high-risk features of PAU were identified in a single-center population of 220 patients who underwent TEVAR from January 2016 to December 2022. Of the patients undergoing TEVAR, 24 patients with PAUs were identified, and those without high-risk features or follow-up imaging were excluded. The inclusion criteria were TEVAR procedure indicated for PAU, at least 1 high-risk radiologic feature associated, and at least 6 months of consistent protocolized imaging follow-up. High-risk radiologic features associated with PAU were defined as associated pleural effusion, periaortic hemorrhage, associated IMH, initial PAU depth >10 mm or neck >20 mm, and total aortic diameter >40 mm. Thus, 12 patients fit the study’s specific, rigorous inclusion criteria.

**Supplementary Figure 1:**
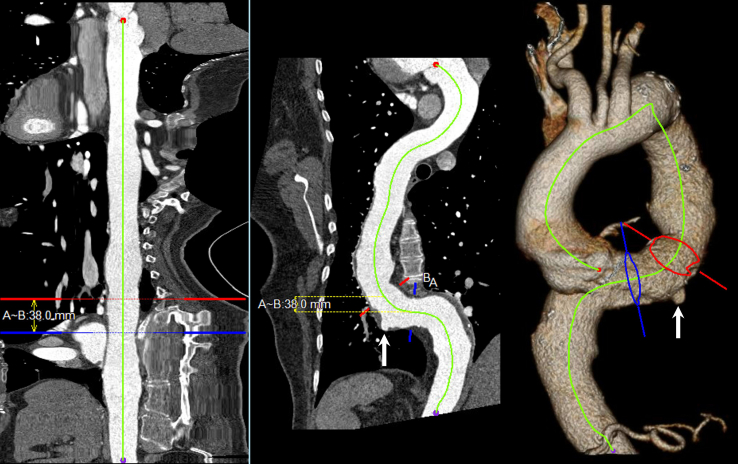


After these 12 patients were identified, demographic, procedural, and postoperative data were collected. Aortic measurements were performed on a dedicated workstation (TeraRecon) with centerline technology. Guided by recent societal reporting standards^1^ and appropriate specific anatomy-related features of PAUs, measurements taken were aortic zone of the PAU, maximum PAU depth perpendicular to the vessel, neck diameter at the point of maximum PAU depth, total aortic diameter at the maximum PAU depth, maximum width of the associated IMH, and length of the IMH hematoma (Supplemental Figure 1).

Aortic diameter 10 mm proximal and distal to the PA and the largest diameter of the aorta in the ascending portion were also measured. These were measured both on the initial computed tomography angiogram and on the most recent follow-up computed tomography angiogram. Results of the remodeling were then determined. The primary outcome was aortic remodeling, and secondary outcomes included PAU thrombosis. Positive aortic remodeling was defined as a decrease in the thickness of the PAU + IMH complex depth >5 mm. Negative aortic remodeling was defined as an increase in the thickness of the PAU + IMH complex >5 mm. If neither occurred, remodeling was deemed stable.

Because of a small number of cases, descriptive statistics were performed on the available variables for the study cohort and reported with a mean ± SD. For nonnormally distributed variables, the median and interquartile range (IQR) were reported. Nonparametric tests, such as Wilcoxon signed rank sum test for distributions in the 2 groups having the same median, were calculated by R Statistical Software (v4.1.2; R Foundation for Statistical Computing).

## Results

The study cohort consisted of 12 patients. Eight (66%) patients were male with a median age of 73 years (IQR, 70.25-78.75 years). Median clinical follow-up was 31 months (IQR, 12.75-59 months), and median imaging follow-up was 12.5 months (IQR, 9.25-26.25 months). Most of the PAUs (7/12 [58%]) were in zone 3, followed by zone 5 (3/12 [25%]).

Four patients (33%) had multiple PAUs, and 9 (75%) of the patients had 3 or more high-risk radiologic factors associated. The most common high-risk feature was associated IMH in 8 (66.7%) patients, followed by maximum aortic diameter >40 mm in 7 (58.3%) patients, initial PAU neck >20 mm in 7 (58.3%) patients, initial PAU depth >10 mm in 6 (50%) patients, periaortic hemorrhage in 6 (50%) patients, and pleural effusion in 4 (33%) patients (Figure; Supplemental Figure 1). Additional demographic and initial PAU characteristics are included in Table 1.

**Figure 1:**
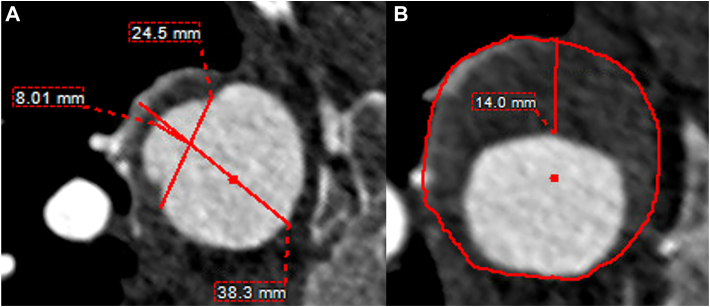
(A) Penetrating aortic ulcer and (B) intramural hematoma measurements.

**Table 1:** Demographic and Initial PAU Characteristics

Age, y	73 (70.25-78.75)
Male	9/12 (66)
Clinical follow-up, mo	31 (12.75-59)
Imaging follow-up, mo	12.5 (9.25-26.25)
Location of PAU	
Aortic zone 2	1 (8)
Aortic zone 3	7 (58)
Aortic zone 4	1 (8)
Aortic zone 5	3 (25)
No. of PAUs	
Single	8 (67)
Multiple	4 (33)
Main PAU depth, mm	10.1 ± 4.5
Main PAU neck, mm	19.5 ± 6.7
IMH associated	8 (67)
IMH thickness, mm	13.7 ± 7.5
IMH length, mm	42.9 ± 26.6
Periaortic hemorrhage associated	6 (50)
Laminar pleural effusion associated	4 (33)
High-risk radiologic features	
1 high-risk feature	1 (8)
2 high-risk features	2 (17)
3 high-risk features	6 (50)
4 high-risk features	2 (17)
5 high-risk features	1 (8)

Categorical variables are presented as number (percentage). Continuous variables are presented as mean ± SD or median (interquartile range).

IMH, intramural hematoma; PAU, penetrating aortic ulcer.

TEVAR was performed with a tube graft in 8 (67%) patients and a tapered configuration in 4 (33%) patients. Proximal graft diameter had a mean of 33.4 ± 3.2 mm, and distal graft diameter had a mean of 32.4 ± 3.5 mm. The most common proximal landing zone was aortic zone 2 in 5 (42%) patients, followed by zone 3 in 4 (33%). The distal aortic landing zone was zone 4 in 8 (67%) and zone 5 in 4 (33%). The left subclavian artery was covered in 7 (57%) patients and revascularized in 5 (41.7%) patients. Procedure characteristics and left subclavian artery revascularization strategies are summarized in Table 2.

**Table 2:** Procedural Characteristics

Characteristic	No. (%) or Mean ± SD
Graft type	
Tube	8 (67)
Tapered	4 (33)
Graft diameter	
Proximal, mm	33.4 ± 3.2
Distal, mm	32.4 ± 3.5
Proximal landing zone	
Aortic zone 1	1 (8)
Aortic zone 2	5 (42)
Aortic zone 3	4 (33)
Aortic zone 4	1 (8)
Aortic zone 5	1 (8)
Distal landing zone	
Aortic zone 4	8 (67)
Aortic zone 5	4 (33)
Total aortic coverage	
50-99 mm	1 (8)
100-149 mm	4 (33)
150-199 mm	3 (25)
200-249 mm	4 (33)
LSA coverage	7 (58)
LSA management	
LCCA to LSA bypass preoperatively	4 (33)
LSA stenting	1 (8)
Conservative	2 (17)

LCCA, left common carotid artery; LSA, left subclavian artery.

**Supplementary Figure 2:**
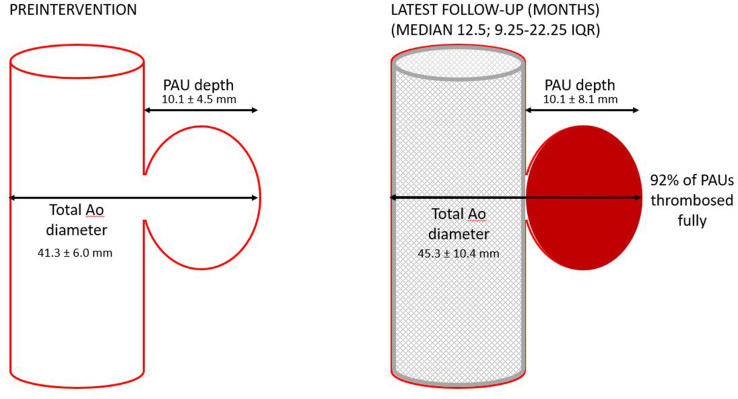


Mean aortic diameter 10 mm proximal to the PAU was 31.3 ± 3.2 mm before intervention and 33.5 ± 3.4 mm at latest follow-up. Mean aortic diameter 10 mm distal to the PAU was 29.5 ± 4.0 mm before intervention and 33.5 ± 3.4 mm at latest follow-up. Main PAU + IMH depth was 10.1 ± 4.5 mm before intervention and 10.1 ± 8.1 mm at the latest follow-up (Supplemental Figure 2). Total aortic diameter was 41.3 ± 6.0 mm before intervention and 45.3 ± 10.4 mm at latest follow-up (Supplemental Figure 3). The main PAU had fully thrombosed in 92% (11/12). Positive aortic remodeling occurred in 25% (3/12), negative aortic remodeling occurred in 17% (2/12), and the aorta remained stable in 58% (7/12) at the latest follow-up (Table 3).

**Table 3:** Penetrating Aortic Ulcer and Aortic Remodeling

Variable	Before Intervention	Latest Follow-Up
Ascending aorta diameter, mm	39.3 ± 2.8	40.4 ± 4.1
Aortic diameter 10 mm proximal to the PAU, mm	31.3 ± 3.2	33.5 ± 3.4
Aortic diameter 10 mm distal to the PAU, mm	29.5 ± 4.0	33.5 ± 3.4
Main PAU+ IMH depth, mm	10.1 ± 4.5	10.1 ± 8.1
Total aortic diameter at PAU, mm	41.3 ± 6.0	45.3 ± 10.4
Positive aortic remodeling		3 (25)
Stable aortic dimensions		7 (58)
Negative aortic remodeling		2 (17)

Categorical variables are presented as number (percentage). Continuous variables are presented as mean ± SD.

IMH, intramural hematoma; PAU, penetrating aortic ulcer.

There were no 30-day deaths. However, 3 (25%) patients died of nonaortic causes during follow-up, all of them at >2.5 years from the procedure (range, 35-56 months). Reinterventions occurred in 17% (2/12) of patients for type 2 endoleak from the left subclavian artery, which was treated with coil embolization, and in another patient who had a new PAU secondary to cocaine abuse.

## Comment

This retrospective cohort study evaluated aortic remodeling in patients with PAUs exhibiting high-risk radiologic features who underwent TEVAR. Despite that PAUs are a known risk factor for adverse remodeling, the data show that TEVAR is associated with either positive remodeling or aortic stabilization in most patients, with favorable short-term safety and efficacy.

Asymptomatic PAUs lacking high-risk characteristics typically have indolent clinical courses and can be safely managed with surveillance and interval imaging. A study of 273 patients demonstrated a low 10-year risk (6.5%) of complications such as rupture or progression in this subgroup.^6^ In contrast, symptomatic PAUs or those associated with IMHs have worse outcomes and are more likely to require intervention. Currently accepted indications for repair include persistent symptoms and radiographic progression in the presence of high-risk features.

The literature on PAU management remains limited, with a low level of evidence guiding current recommendations.^5^^,^^8^ This highlights the need for larger, prospective, multicenter studies to define natural history and optimal timing for intervention. After TEVAR, aortic remodeling and healing dynamics are important to understand. This study suggests that the aorta tends to conform to the diameter of the stent graft, helping stabilize the diseased segment and reduce the risk of future aneurysmal degeneration. Most treated PAUs fully thrombosed after intervention because of flow exclusion.

Most of our patients experienced positive remodeling, defined as a >5-mm reduction in the PAU + IMH complex, and outcomes were favorable, with no 30-day mortality and 100% 2-year survival. Importantly, none of the 3 late deaths were aorta related, further supporting TEVAR's safety in this high-risk group.

Limitations include retrospective design, selection bias, and lack of standardized definitions for aortic remodeling in the context of PAUs. Nevertheless, the findings contribute to a growing body of evidence supporting early intervention in select high-risk patients.

In conclusion, TEVAR offers a safe and effective treatment strategy for PAUs with high-risk features, leading to positive or stable aortic remodeling in most patients. Aorta-related complications and reintervention rates are low. Further studies are needed to refine selection criteria and to understand long-term outcomes in this rare but high-stakes disease entity.

## Figures and Tables

**Supplementary Figure 3:**