# Supplementary material for: Aortic Remodeling in Patients Undergoing Endovascular Repair for Penetrating Aortic Ulcer With High-Risk Features
Source: Ann Thorac Surg Short Rep. 2025 Jul 29;4(1):17–21. doi: 10.1016/j.atssr.2025.07.001 (PMC13100723; doi:10.1016/j.atssr.2025.07.001)
Supplement: Supplementary Figures Legend [file mmc1.docx]

Supplemental Figure 1. Length and Centerline of PAU and IMH

Supplemental Figure 2. Aortic diameters and PAU depth (Supplemental Material)

Supplemental Figure 3. Aortic diameters proximal and distal to PAU (Supplemental Material)
